# Polymerizable Microsphere-Induced High Mechanical Strength of Hydrogel Composed of Acrylamide

**DOI:** 10.3390/ma11060880

**Published:** 2018-05-24

**Authors:** Zhiyong Wang, Meiqin Lin, Menghan Wang, Xia Song, Chuqiao Zhang, Zhaoxia Dong, Juan Zhang, Zihao Yang

**Affiliations:** Research Institute of Enhanced Oil Recovery, China University of Petroleum, Beijing 102249, China; zoyicup@126.com (Z.W.); wmhh92@126.com (M.W.); songxiasummer@163.com (X.S.); zhangchuqiao0108@163.com (C.Z.); dzx@cup.edu.cn (Z.D.); juanzhang@cup.edu.cn (J.Z.); zihaoyang@cup.edu.cn (Z.Y.)

**Keywords:** composite hydrogel, three-dimensional pore structure, tensile property, compression strength, rheological properties

## Abstract

Polymerizable microspheres are introduced into acrylamide to prepare the high mechanical strength hydrogels with a novel three-dimensional pore structure. Rheological properties, compressive stress–strain, tensile property, and compression strength of three different types of hydrogels were investigated. Moreover, a scanning electron microscope (SEM) was adopted to observe the three-dimension network structure of three different types of hydrogels. The test results illustrated that viscous moduli (G″) and elastic moduli (G′) of a hydrogel containing polymerizable microspheres (P) reached maximum values, compared to the normal hydrogel (N) and the composite hydrogel containing ordinary microspheres (O). When the hydrogels were squeezed, the N was easily fractured under high strain (99%), whereas the P was not broken, and quickly recovered its initial morphology after the release of load. The P showed excellent tensile properties, with an elongation at break up to 90% and a tensile strength greater than 220 g. The compression strength of the N was 100.44 kPa·m^−1^, while the resulting strength of P was enhanced to be 248.00 kPa·m^−1^. Therefore, the various performances of N were improved by adding polymerizable microspheres. In addition, the SEM images indicated that N has a general three-dimensional network structure; the conventional network structure did not exist in the P, which has a novel three-dimensional pore structure in the spherical projection and very dense channels, which led to the compaction of the space between the three-dimensional pore network layers and reduced the flowing of free water wrapped in the network. Therefore, the mechanical strength of hydrogel was enhanced.

## 1. Introduction

Because of their unique structure and physicochemical characters, polymer hydrogel materials have a wide application prospect in the fields of petroleum drilling [1], flexible chemical mechanical devices [2], selective filtration [3,4], biomedicine [5,6,7], and tissue engineering [8,9]. However, the mechanical properties of the traditional chemical crosslinked hydrogels are poor, which limits their application in many fields. In order to improve their mechanical properties, a series of new hydrogels with excellent mechanical properties have been developed. Because of their simple preparation methods and excellent mechanical properties in particular, composite hydrogels have attracted wide attention from researchers.

A new polymer/attaplugite nano-composite hydrogel with improved response rate and tensile mechanical was reported, but the mechanical properties of this material are also very general; for example, the maximum tensile stress of the optimum sample with the attaplugite content of 0.05% is only about 35 kPa [10]. Similarly, Haraguchi introduced clay (6.2%) into the monomer to improve the mechanical strength of the hydrogel. The composite hydrogel exhibits a monotonic increase in stress with increasing strain, with a tensile stress of 109 kPa [11,12,13]. Nanocomposite hydrogels consisting of inorganic (silica nanoparticles)/organic (poly (acrylic acid)) (SNP) network structures are synthesized by in-situ free polymerization from the SNP surface in an acrylic acid (AA) aqueous solution. The maximum tensile stress of this material with the silica content of 0.05% was about 110 kPa [14]. It can be seen that the mechanical strength of the gel is limited by physical crosslinking.

Using nano-microspheres as the crosslinking point of hydrogels is an effective method to synthesize functional hydrogels with excellent mechanical properties [15,16]. However, the synthesis of the hydrogels by using nano-microspheres (NC gels) as the crosslinking point requires a very careful and complex surface structure design [17,18,19]. Compared to the traditional chemical cross-linking hydrogels (OR gels), the mechanical properties of NC gels have been improved greatly. Furthermore, the swelling property and optical transmissibility have also been improved. As a result, NC gels are considered to almost solve all defects existing in OR gels [20,21,22]. However, due to the characteristics of NC gels’ network structure, it is difficult to achieve its functional construction. In order to solve this problem, macromolecular microspheres composite hydrogels (MMC) have been developed. Hu synthesized MMC samples by introducing hydroxyl and carboxyl groups on the microspheres surface, then the adjacent microspheres crosslinked by divinyl alum and epoxy chloropropane [23,24]. This hydrogel has fast response speed, and the color of hydrogels will change as the size and concentration of different microsphere, but there is no neat macro network structure in the hydrogel. Morimoto first reported a macromolecule containing methacrylic groups on the surface and synthesized micro hydrogels with Prussian blue as the template; then, the monomer grew on the surface and synthesized the hydrogels, using the micro hydrogel as the crosslinking point [25]. The hydrogel showed an excellent ability to bind proteins, but the synthesis process of the hydrogel was complex, the process was strict, and the yield was low. Macromolecular microspheres were irradiated using ^60^Co γ-rays, and the active free radicals were produced on the surface; then further polymerization of the acrylic monomer on the microsphere surface occurred [26]. The synthesis process was relatively complex, and ^60^Co will release a lot of heat in the polymerization process, so it is not conducive to the polymerization of N-isopropyl acrylamide. In addition, the ^60^Co γ-rays radiation equipment requires a rigorous experimental condition. Xia proposed a novel strategy to improve stretchability, fatigue resistance, and self-recoverable properties of hydrogels by using a SiO_2_-g-poly (butyl acrylate) core–inorganic and shell–organic hybrid latex particles as hydrophobic crosslinking centers for hydrophobic association [27]. A series of chemically cross-linked microgel composite hydrogels (MCH gels) with excellent toughness and stretchability were prepared, using core-shell polymer microspheres as cross-linking junctions [28]. However, the high tensile properties of the hydrogel as a killing fluid are far from meeting the requirements of the application, and the viscoelastic and compressive properties of the samples are also very important.

**Scheme 1:**
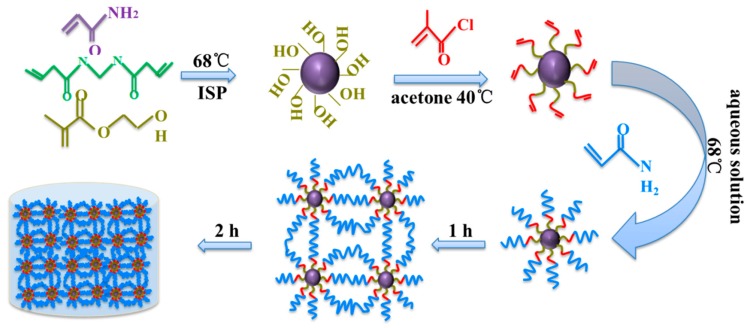
The preparation process of microsphere hydrogels (ISP: inverse suspension polymerization).

In this study, we propose a novel strategy to prepare high mechanical strength hydrogels with a novel three-dimensional pore structure, by introducing polymerizable microspheres as cross-linking centers. As shown in the Scheme 1, there are a lot of hydroxyl groups on the surface of copolymer microspheres with incorporated acrylamide (AM) and 2-hydroxyethyl methylacrylate (HEMA) (Figure S1a). The reaction of methacryloyl chloride with the hydroxyl groups was performed; as a result, the polymerizable double bond (C=C) was introduced on the surface of the AM/HEMA microspheres, as shown in Figure S1b in the Supporting Information. Subsequently, the polymerizable microspheres were dispersed in acrylamide solution, and the free radical polymerization of acrylamide and polymerizable microspheres under the action of initiator potassium persulfate (KPS) formed the dense three-dimensional pore structure. A lot of compact three-dimensional pore structures were formed between microspheres. Compared with the normal hydrogels (N) and the ordinary microsphere hydrogels (O), the polymerizable microsphere hydrogels with three-dimensional pore structure (P) have higher rheological properties, compressive performance, and tensile properties, which will be potentially used in the petrochemical industry.

## 2. Experiment

### 2.1. Material

The monomers acrylamide (AM, 99%), 2-hydroxyethyl methacrylate (HEMA, 98%), *N*, *N*-dimethyl ethanolamine (DMEA, 99%), and meth acryloyl chloride (96%) were purchased from Shanghai Macklin Biochemical Co., Ltd. (Shanghai, China). *N*, *N*′-methylene bis acrylamide (MBA, 99%) was used as a crosslinking agent, potassium persulfate (KPS) was used as the initiator, and Span 80 (AR) was used as an emulsifier—all were purchased from the Tianjin Kwang Fu Fine Chemical Industry Research Institute (Tianjin, China). Anhydrous sodium carbonate was purchased from the Qinhuangdao Chemical Reagent Factory. Tenth white oil (industrial grade) was purchased from Fushun Sunway Petrochemical Co., Ltd. (Fushun, China). Acetone (AR) and absolute ethanol (99.5%) used as a solvent was purchased from Beijing Chemical Factory (Beijing, China).

### 2.2. Synthesis of Acrylamide/2-Hydroxyethyl Methacrylate Microspheres

We synthesized the composite microspheres by inverse suspension polymerization at a low temperature. Firstly, about 3 g Span 80 and 80 g 10th white oil were added into a three-neck round-bottom flask, which was heated to 50 °C in the water bath and stirred adequately at the speed of 250 rpm for 0.5 h. Then a certain amount of AM (12 g), HEMA (8 g), MBA (0.1 g), KPS (0.2 g), and deionized water were added to the beaker and stirred at room temperature for 0.5 h. Next the water phase was added to the oil phase, at the speed of two drops per second at 18 °C; at the same time, the mixture was emulsified at the speed of 350 rpm for 0.5 h. Subsequently, DMEA was added into the flask, which was stirred continually for 1.5 h. At last, The resulting reaction solution was poured into the centrifugation tubes, and the polymer microspheres were collected by centrifugation and washed with absolute ethanol several times to remove the copper catalyst; these were then dried at 50 °C for 24 h under vacuum (−0.09 MPa) to provide a white powder. The yield of these polymer microspheres is about 99%.

### 2.3. Treatment of the Acrylamide/2-Hydroxyethyl Methacrylate Microspheres

Into the flask were added 2.00 g AM/HEMA composite microspheres and 40 mL acetone as the solvent. After stirring for 0.5 h at the speed of 300 rpm at 40 °C, 5.00 mL methacryloyl chloride was added to the flask, which was stirred continuously for another 0.5 h, after which 0.05 g anhydrous sodium carbonate was added; this mixture reacted for 12 h. The resulting reaction solution was poured into the centrifugation tubes, and the polymer microspheres were collected by centrifugation and washed with absolute ethanol four times, which were then dried at 50 °C for 24 h under vacuum (−0.09 MPa) to provide a white powder. The yield of these polymer microspheres was about 98%.

### 2.4. Preparation of the Microsphere Hydrogel

2.00 g microspheres were added to the 92.00 g deionized water, after swelling for 12 h, 6.00 g (or 8.00 g) AM were added to the solution, which were stirred for 0.5 h. Then a certain amount of MBA (0.04 g) and KPS (0.05 g) were added into the solution, continuing to stir for 4 h at a speed of 350 rpm. In order to remove the oxygen, nitrogen was introduced into the solution for 30 min. Finally, the beaker was sealed with plastic wrap and stood in the 68 °C ovens. The formation and polymerization of the microspheres, as well as the mechanism for the formation of a hydrogel, were proposed in Scheme 1.

### 2.5. Rheological Measurements

The rheological properties of hydrogels were measured by using a HAAKE RS600 rheometer (German HAAKE Company, Berlin, Germany). The measurement sensor was PP20 plate-to-plate geometry with a gap of 1 mm. The hydrogel samples, with a diameter of 10 mm and thickness of 2 mm, were prepared by using a polytetrafluoroethylene mold. The measurements were initially set in a stress oscillation scan mode at the stress range of 0~250 Pa and a sheer frequency of 0.5 Hz to obtain the scope of linear viscoelasticity, in which subsequent frequency-sweeping tests were performed at a fixed frequency of 0.5 Hz to achieve viscous modulus (G″) and elastic modulus (G′) curves as the function of time. The tests were all carried out at 25 °C.

### 2.6. Strain–Stress Measurements

Strain–stress tests were performed to measure the compressive strength at an ambient temperature using the TA.XT PLUS texture analyzer (Lotun Science Co., Ltd., London, UK). The cylindrical gel samples were made of a polytetrafluoroethylene mold with a diameter of 15 mm and thickness of 15 mm. In the experiment, the constant load of 50 kg was moved to compress the cylindrical samples at a rate of 2 mm·min^−1^, the compressed strain of the samples was set to 90% and 99%, and the speed of return after compression was 10 mm·s^−1^.

### 2.7. Tensile Test

Tensile tests were carried out on the TA.XT PLUS texture analyzer. The dumbbell mold was made of polytetrafluoroethylene, and the effective length of the gel was 2 cm. Debugging device and gel sample, when the 5 g force was induced, it began to trigger. The drawing speed before the trigger was 1 mm·min^−1^, and the drawing speed was 3 mm·min^−1^ after the trigger.

### 2.8. Compression Strength Measurements

The compression strength tests device was used to estimate the mechanical strength of hydrogels [1]. A microsphere composite crosslinking solution was mixed into the visible organic glass device, and then placed in a 70 °C constant temperature and gelatinized completely. After the crosslinking solution in the steel casing gelatinized, the compressive properties of hydrogel were measured in such a way that nitrogen gas from the nitrogen cylinder was aerated through the bottom of casing to pressurize the hydrogel, so as to measure the hydrogel’s breakthrough pressure.

### 2.9. Scanning Electron Microscopy (SEM)

The hydrogel samples were frozen sufficiently in liquid nitrogen. The fresh hydrogel samples were rapidly plunged into liquid nitrogen for about 15 min, and then freeze-dried for 24 h to remove water. The freeze-dried hydrogel samples were broken into small pieces, and the fresh surfaces were investigated with a quanta 200F scanning electron microscopy (SEM) (American FEI Company, Hillsboro, AL, USA). Before SEM investigation, a thin layer of gold was sputter-coated.

## 3. Results and Discussion

### 3.1. Rheological Behavior of Microsphere Hydrogel

**Figure 1:**
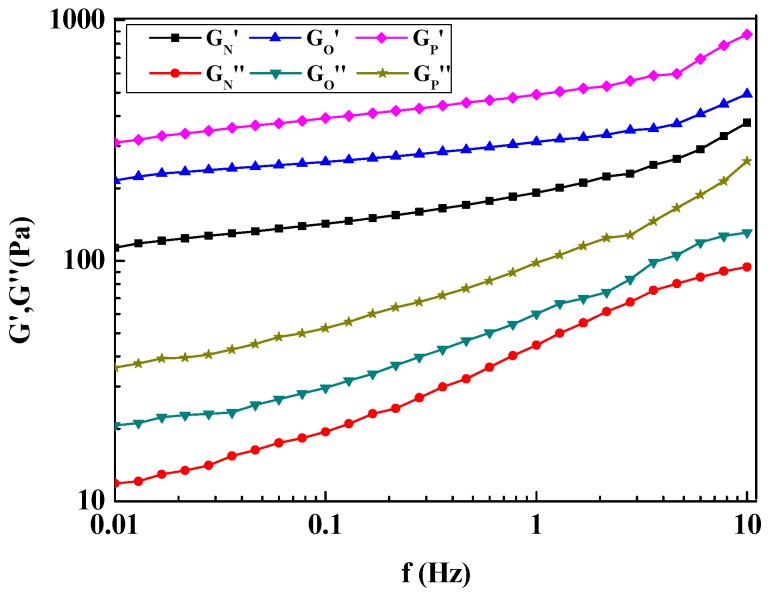
Viscous moduli (G″) and elastic moduli (G) of hydrogels: normal hydrogel (G_N_), hydrogel containing ordinary microspheres (G_O_), and hydrogel containing polymerizable microspheres (G_P_).

The effects of ordinary and polymerizable microspheres on the viscous moduli (G″) and elastic moduli (G′) of hydrogels were shown in Figure 1. G″ and G′ significantly increased after adding microspheres, indicating that strong interactions exist between polymer chains and spherical particles. However, the hydrogels with different microspheres varied dramatically in G″ and G′ values. The maximal G_N_″ and G_N_′ of a normal hydrogel were 94.21 and 375.20 Pa, respectively. Obviously, when the polymerizable microspheres were added, the G″ and G′ of the hydrogels were significantly improved. The maximum G″ and G′ values of the composite gel containing ordinary microspheres were 131.24 and 493.54 Pa, which could be explained by the increasing friction among polymer chains in a more compact network structure, because of the ordinary microspheres mosaic in the network skeletons. When the ordinary microspheres were replaced by the polymerizable microspheres, the maximum G″ and G′ values of the hydrogels were 874.30 and 259.66 Pa, increasing by 175.62% and 133.02%, respectively, compared to the normal hydrogel. This is because the polymerizable microspheres have C=C bonds, so free radical polymerization can be carried out with the AM monomer under the action of the initiator (KPS); many dense meshes will form on the surface of the polymerizable microspheres, so the mechanical strength of the gel has been greatly improved. Due to the swollen ordinary microspheres and the hydrogel network skeleton tightly adhered together, when the hydrogels were sheared, the viscous resistance between shearing layers increased, decreasing the fluidity of free water wrapped in the network. The difference is that, compared with the physical adhesion between the ordinary microspheres and the hydrogel skeleton, the adhesion between the polymerizable microspheres and the hydrogel is greater, which is caused by the chemical reaction. There can be no doubt that the expansible polymerizable microspheres played an important role in improving the strength of the hydrogels.

In addition, polymerizable microspheres contributed more to the G″ and G′ than ordinary microspheres; with G” increasing nearly one times than that of G′, this may be attributed to the rigidity and smoothness of polymerizable microspheres. As shown in Figure 1, G′ remained larger than G″ over the varying types of polymer microspheres, and the differences between G″ and G′ increased progressively as frequency increased, indicating that microsphere hydrogels are viscoelastic solids (G′ > G″). In addition, for both normal and microsphere hydrogels, in the small frequency range (0.01–0.1 Hz, logarithmic coordinate), G′ was frequency-independent, and three testing curves G_N_′, G_O_′, and G_P_′ change little with frequency—whereas in a large frequency range (0.1–10 Hz, logarithmic coordinate), G′ increased with frequency. In the whole frequency range (0.01–10 Hz), G″ of the P sample increased more sharply than that of the hydrogel with ordinary microspheres (O) and normal hydrogel. The different frequency dependence for both modules was attributed to the change of network structure formed by polyacrylamide and a crosslinking agent with different polymer microspheres. As G″ and G′ reflect the adhesion and strength of hydrogel, the larger the G″ and G′, the stronger the hydrogel’s abilities to resist compression and deformation. The P sample has better mechanical properties.

### 3.2. Strain–Stress Properties of Microsphere Hydrogels

**Figure 2:**
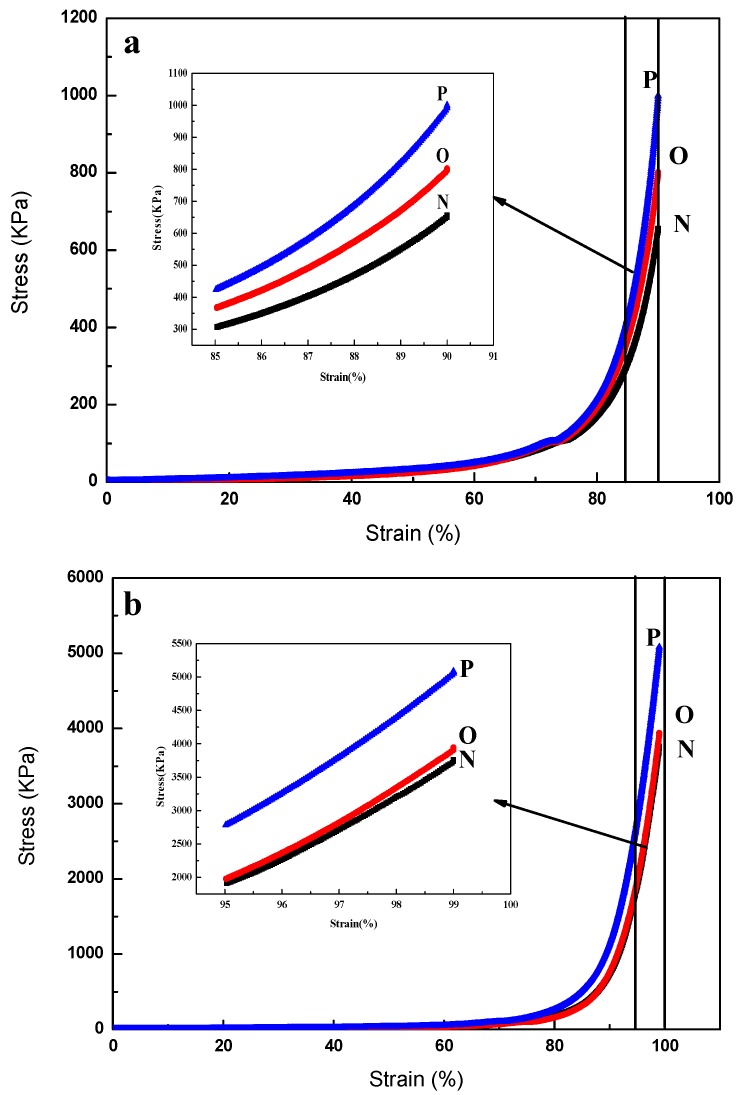
Strain–force curves of hydrogels: normal hydrogel (N), hydrogel containing ordinary microspheres (O), and hydrogel containing polymerizable microspheres (P). (**a**) Compression strain 90%; (**b**) compression strain 99%.

The mechanical properties of normal and microsphere hydrogels can be reflected by using compressive stress–strain tests. As shown in Figure 2, when the hydrogel samples were compressed, they will present elastic deformation and store energy to resist the applied load. The order of the capacity of load resistance for the hydrogel sample is P > O > N. Taking compression strain of 90% as an example, the stress of the P sample, O sample, and the normal sample were 1000, 800, and 655 kPa, respectively. In addition, the normal hydrogel is more fragile; because of its lower elastic modulus, it is easy to break. However, the microsphere hydrogels cannot be broken under high strain, and they can recover their original shape after releasing pressure, which is because the polymer microspheres have good elasticity after swelled. It can be found from Figure 2 that the stress increased with the increase of compressive strain. In the low strain region (0–20%), the hydrogel stress was nearly in parallel with strain, which indicated that the gel samples have very little elastic deformation and less energy storage. In the medium strain region (20–80%), the stress increased obviously with the increase of strain. The rate of increasing of the P sample was the fastest, and the rate of the microsphere hydrogels was slightly higher than that of the normal hydrogel. The reason for this may be that the existence of the adhesion between microspheres and hydrogels matrix increased the resistance ability of the microsphere hydrogels. The framework of the normal hydrogel was thin, the pores of the O sample were dense, and the skeleton was very thin. In the high strain region (80–90%), the stress increased sharply, indicating that the hydrogels stored enough energy to resist compression. As the compression continued, the three-dimensional pore network structure of the P sample became more and more compact with the increasing strain, and the flow friction between structure layers remained high, owing to the dispersion of polymerizable microspheres. Meanwhile, polymer chains came close to their full extension, which led to the increasing elastic deformation and energy storage, so the stress was induced to increase rapidly.

**Figure 3:**
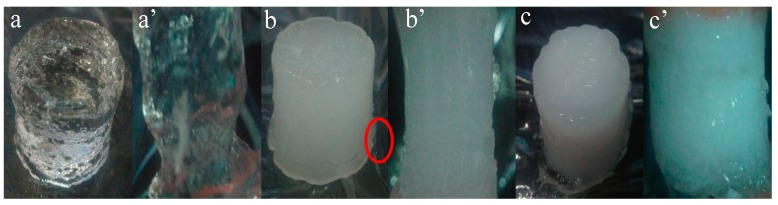
Morphological contrast between original samples and compressed samples (compression strain 99%); (**a**) N, (**a’**) compressed N; (**b**) O, (**b’**) compressed O; (**c**) P, (**c’**) compressed P.

It can be clearly observed from the Figure 3a’,b’) that, the N sample has been broken, while the O sample has a slight crack. However, there was no change in the P sample (Figure 3c,c’), which can restore its original state and also not show obvious traces of fracture after a slight stretch. The results show that the compression performance of the hydrogel containing the polymerizable microspheres is better than that of the other two hydrogels.

### 3.3. Tensile Properties of Microsphere Hydrogels

**Figure 4:**
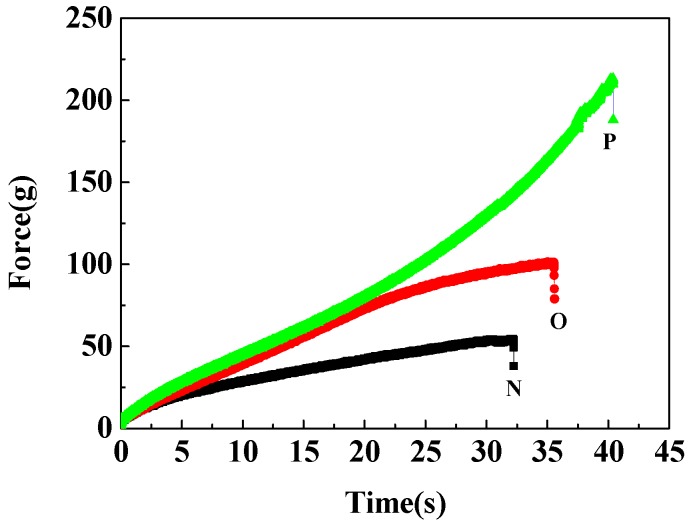
Stretching curves of hydrogels: normal hydrogel (N), hydrogel containing ordinary microspheres (O), and hydrogel containing polymerizable microspheres (P).

The typical stretch curves of hydrogel samples are shown in Figure 4. It is found that the tensile property of the normal gel is the worst, and the P sample has the best tensile property. The longest stretching times of three different gels were 32, 35, and 40 s, the maximum tensile force were 35, 75, and 220 g, respectively. The highest tensile strength was achieved in P hydrogel sample, which could be explained by the proposed three-dimensional pore network structure. Since the polymer chains connecting polymerizable microspheres were long and flexible, in order to be extended to a large extent on macroscopic deformation, the O sample can be greatly deformed without damaging the constituent polymer chains. The main reason is that a special three-dimensional pore honeycomb network structure was formed between the polymerizable microspheres and AM.

**Figure 5:**
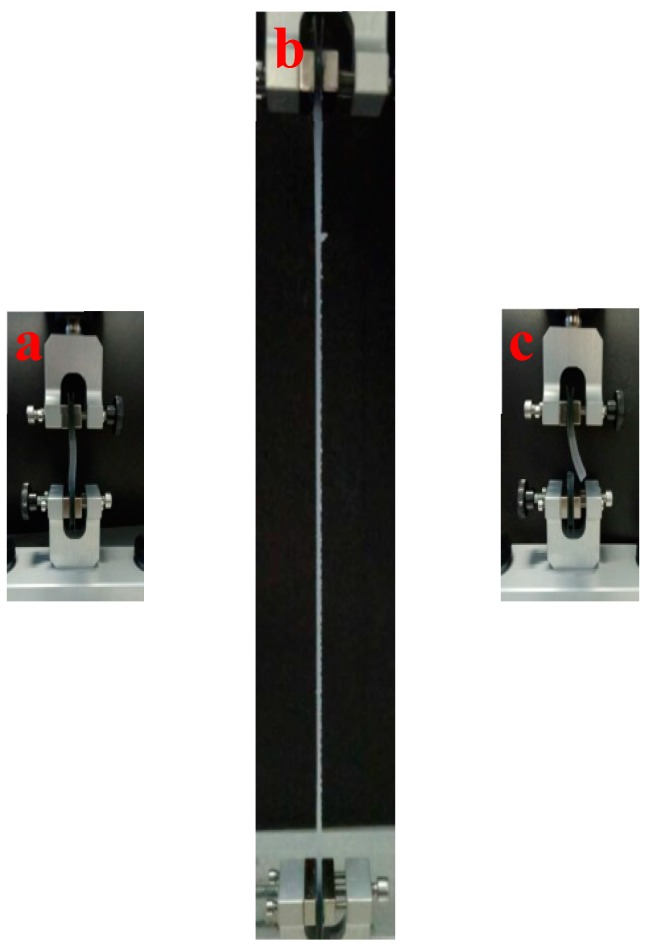
Tensile drawing of the hydrogel sample containing polymerizable microspheres. (**a**) Length: 2 cm, diameter: 1 cm; (**b**) length: 20 cm, diameter: 0.25 mm; (**c**) sample fractured.

Figure 5 shows a typical tensile process for the P hydrogel sample over a wide range. It can be seen that the final fracture of the P hydrogel sample was at the end of the dumbbell. However, both N and O hydrogel samples were fractured in the intermediate part. It may be that the actual tensile properties of the P hydrogel sample were better. This result can be explained by the fact taht in the case of the hydrogel-containing polymerizable microspheres, the probability of formation of a cross-linked chain between the polymerizable microspheres and AM increased, due to the effective conversion of graft or looped chains into effective cross-linking chains and the decreased inter-particle distance. In summary, the hydrogel containing polymerizable microspheres exhibited the best tensile properties, which were reflected in subsequent compression and pressure tests.

### 3.4. Compression Strength of Microsphere Hydrogel

Compression strength measurements were completed using compression strength tests device self-designed in the laboratory [1]. In the initial measurement period, when the nitrogen pressure applied increased, three different types of hydrogels showed perfect resistance to the increasing nitrogen pressure and did not break. When the gas pressure continued to rise to higher values, the hydrogels also maintained their initial state. Nevertheless, a few minutes later gas channeling at the wall of the visible organic glass casing or the hydrogels showed bubbling from the bottom up; then the gas pressure value quickly reduced to nearly zero, which made it clear that the hydrogels were cracked through by this high applied gas pressure. The compression strength measured was determined by both its strength and the adhesion between hydrogel and wall of the casing. When the hydrogel strength was weak, high nitrogen pressure could have led to the hydrogel cracking in the form of bubbling, caused by the elasticity of hydrogel. However, if the adhesion between hydrogel and casing was low, high pressure will force nitrogen gas to flow out from the gap that existed between hydrogel and casing. In view of this, a sufficiently high hydrogel breakthrough pressure can be defined as the compressive strength of the hydrogels. Therefore, this test was an effective method to measure the compression strength of hydrogel to evaluate its application for well killing.

In this experiment, the normal hydrogel was broken through easily, with the nitrogen pressure increasing, whereas the microsphere hydrogels can resist the gas pressure and maintain themselves in original morphology. Increasing nitrogen pressure was forced on microsphere hydrogel from the bottom up, which was equivalent to the formation pressure imposed on the microsphere hydrogel as killing fluids during the process of well killing. In view of the failure of the hydrogel in the compression strength test, which mainly resulted from its weak mechanical strength, microsphere particles were introduced, to be added into the polymer crosslinking system. This not only increased the hydrogel’s compressive strength, but also increased the adhesion between hydrogel and wall of the casing. The effects of different types of microspheres on the compression strength of hydrogel are shown in Table 1. The compression strength of normal hydrogel was 100.44 kPa·m^−1^, whereas the compression strength of the O and P samples were 202.45 and 248.00 kPa·m^−1^, respectively, which demonstrates that the compression strength of hydrogel was obviously increased. In short, this increase is due to the chemical reaction between polymerizable microspheres and AM, which formed the special three-dimensional pore net structure.

### 3.5. Morphology and Gelling Mechanism of Hydrogels

**Figure 6:**
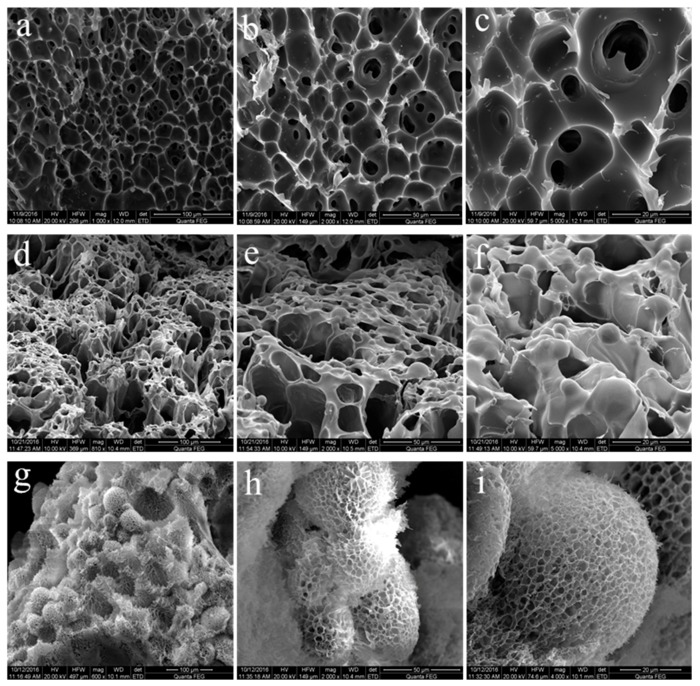
SEM images of hydrogels: (**a**–**c**) normal hydrogel; (**d**–**f**) hydrogel containing ordinary microspheres; and (**g**–**i**) hydrogel containing polymerizable microspheres.

The SEM images of normal hydrogel, hydrogel containing ordinary microspheres, and hydrogel containing polymerizable microspheres with different magnification are shown in Figure 6. It can be observed that there are obviously different surface morphologies between the three different types of hydrogels. The conventional hydrogel network structure can only be observed in SEM images (normal hydrogel samples, Figure 6a–c); the pore distribution is more uniform, the channels are often connected with each other, and some open channels are formed. In the SEM images of the O samples (Figure 6d–f), the conventional hydrogel network structure can be observed; at the same time, it can be observed that it is spherically convex—that is, the ordinary microspheres are contained in the hydrogel skeleton, and the distribution of ordinary microspheres is uniform. Therefore, the thickness of the hole walls was increased due to the presence of the ordinary microspheres. The conventional network structure did not exist in the P samples (Figure 6g–i); their hydrogel network structure is similar to the honeycomb in the spherical projection, and the channels are very dense. This is because the double bonds (C=C) exist on the surface of polymerizable microspheres (Figures S2 and S3), which polymerize with the acrylamide and form a three-dimensional pore structure. This is because the three-dimensional pore structure is very strong, so the hydrogel containing the polymerizable microspheres has a high mechanical strength, which corresponds to the test results mentioned above.

## 4. Conclusions

Because of the addition of polymerizable microspheres, the skeleton and network structure of the hydrogels were changed, and the P sample showed high mechanical strength. Compared to the normal hydrogel, both G″ and G′ of microsphere hydrogels were obviously improved. The normal hydrogel was easily fractured under high strain (99%), whereas the polymerizable microsphere hydrogels were not broken, and can quickly recover their original shape after the release of load and improve the tensile properties. The compression strength of hydrogel was increased from 100.44 to 248.00 kPa·m^−1^, due to the improved strength and adhesion induced by polymerizable microspheres. The novel three-dimensional pore network structure was compacted and the fluidity of free water wrapped in the network was limited, owing to the interaction between microspheres and PAM, which contributed to the high mechanical properties of polymerizable microsphere hydrogel. Our study could inspire more future research on the polymerizable microsphere hydrogel for well-killing application in the reservoir.

## Figures and Tables

**Table 1:** Compression strength of hydrogels with different types of polymer microspheres.

Hydrogel Sample	AM % (Mass Fraction)	Polymeric Microsphere % (Mass Fraction)	MBA % (Mass Fraction)	Compression Strength (kPa·m^−1^)
N sample	8	0	0.05	100.44
O sample	6	2	0.05	202.45
P sample	6	2	0.05	248.00

## Supplementary Materials

The following are available online at http://www.mdpi.com/1996-1944/11/6/880/s1, Figure S1: SEM images of acrylamide (AM)/2-hydroxyethyl methylacrylate (HEMA) microspheres (a) ordinary polymeric microspheres; (b) polymerizable microspheres, Figure S2: FT-IR spectra of polymer (a) polymerizable AM/HEMA microspheres; (b) hydrogel containing polymerizable microspheres, Figure S3: ^13^C NMR spectrum of polymer (a) polymerizable AM/HEMA microspheres; (b) hydrogel containing polymerizable microspheres.

## References

[1] Sun F., Lin M., Dong Z., Zhang J., Wang C., Wang S., Song F. (2015). Nanosilica-induced high mechanical strength of nanocomposite hydrogel for killing fluids. J. Colloid Interface Sci..

[2] Sangwan W., Petcharoen K., Paradee N., Lerdwijitjarud W., Sirivat A. (2016). Electrically responsive materials based on polycarbazole/sodium alginate hydrogel blend for soft and flexible actuator application. Carbohyd. Polym..

[3] Wu S., Braschler T., Anker R., Wildhaber F., Bertsch A., Brugger J., Renaud P. (2015). Composite hydrogel-loaded alumina membranes for nanofluidic molecular filtration. J. Membr. Sci..

[4] Zhao K., Zhang X., Wei J., Li J., Zhou X., Liu D., Liu Z., Li J. (2015). Calcium alginate hydrogel filtration membrane with excellent anti-fouling property and controlled separation performance. J. Membr. Sci..

[5] Zhang Y., Tao L., Shuxi L., Wei Y. (2011). Synthesis of Multiresponsive and Dynamic Chitosan-Based Hydrogels for Controlled Release of Bioactive Molecules. Biomacromolecules.

[6] Xu X., Xu Z., Yang X., He Y., Lin R. (2017). Construction and characterization of a pure protein hydrogel for drug delivery application. Int. J. Biol. Marcomol..

[7] Singh B., Sharma V. (2017). Crosslinking of poly(vinylpyrrolidone/acrylic acid with tragacanth gum for hydrogels formation for use in drug delivery applications. Carbohyd. Polym..

[8] Wang J., Zhang F., Tsang W.P., Wan C., Wu C. (2017). Fabrication of injectable high strength hydrogel based on 4-arm star PEG for cartilage tissue engineering. Biomaterials.

[9] Fan M., Ma Y., Tan H., Jia Y., Zou S., Guo S., Zhao M., Huang H., Ling Z., Chen Y. (2017). Covalent and injectable chitosan-chondroitin sulfate hydrogels embedded with chitosan microspheres for drug delivery and tissue engineering. Mater. Sci. Eng. C.

[10] Xiang Y., Peng Z., Chen D. (2006). A new polymer/clay nano-composite hydrogel with improved response rate and tensile mechanical properties. Eur. Polym. J..

[11] Haraguchi K., Takehisa T. (2002). Nanocomposite hydrogels: A unique organic–inorganic network structure with extraordinary mechanical, optical, and swelling/de-swelling properties. Adv. Mater..

[12] Haraguchi K., Takehisa A.T., Fan S. (2002). Effects of clay content on the properties of nanocomposite hydrogels composed of poly(*N*-isopropylacrylamide) and clay. Macromolecules.

[13] Haraguchi K., Li H.J. (2006). Mechanical properties and structure of polymer−clay nanocomposite gels with high clay content. Macromolecules.

[14] Yang J., Zhao J. (2014). Preparation and mechanical properties of silica nanoparticles reinforced composite hydrogels. Mater. Lett..

[15] Zheng Y., Ji S., Liu H., Li M., Yang H. (2012). Synthesis of mesoporous γ-AlOOH@Fe_3_O_4_ magnetic nanomicrospheres. Particuology.

[16] Zheng L., Pi F., Wang Y., Xu H., Zhang Y., Sun X. (2016). Photocatalytic degradation of Acephate, Omethoate, and Methyl parathion by Fe_3_O_4_@SiO_2_@mTiO_2_ nanomicrospheres. J. Hazard. Mater..

[17] Zou S., Liu H., Yang Y., Wei Z., Wang C. (2013). Multihollow nanocomposite microspheres with tunable pore structures by templating Pickering double emulsions. React. Funct. Polym..

[18] Hazan Y.D., Märkl V., Heinecke J., Aneziris C., Graule T. (2011). Functional ceramic and nanocomposite fibers, cellular articles and microspheres via radiation curable colloidal dispersions. J. Eur. Ceram. Soc..

[19] Wang C., Zhang C., Li Y., Chen Y., Tong Z. (2009). Facile fabrication of nanocomposite microspheres with polymer cores and magnetic shells by Pickering suspension polymerization. React. Funct. Polym..

[20] Haraguchi K., Li H.J., Matsuda K., Takehisa T., Elliott E. (2005). Mechanism of forming organic/inorganic network structures during in-situ free-radical polymerization in PNIPA−clay nanocomposite hydrogels. Macromolecules.

[21] Xiong L., Zhu M., Hu X., Liu X., Tong Z. (2009). Ultrahigh deformability and transparence of hectorite clay nanocomposite hydrogels with nimble pH response. Macromolecules.

[22] Nie J., Du B., Oppermann W. (2004). Influence of formation conditions on spatial inhomogeneities in poly(*N*-isopropylacrylamide. Hydrogels. Macromolecules.

[23] Hu Z., Lu X., Gao J., Wang C. (2000). Polymer gel nanoparticle networks. Adv. Mater..

[24] Hu Z., Lu X., Gao J. (2001). Hydrogel Opals. Adv. Mater..

[25] Morimoto N., Endo T., Iwasaki Y., Akiyoshi K. (2005). Design of hybrid hydrogels with self-assembled nanogels as cross-linkers: interaction with proteins and chaperone-like activity. Biomacromolecules.

[26] Huang T., Xu H.G., Jiao K.X., Zhu L.P., Brown H.R., Wang H.L. (2007). A novel hydrogel with high mechanical strength: A macromolecular microsphere composite hydrogel. Adv. Mater..

[27] Xia S., Song S., Ren X., Gao G. (2017). Highly tough, anti-fatigue and rapidly self-recoverable hydrogels reinforced by core-shell inorganic-organic hybrid latex particles. Soft Matter.

[28] Xu K., Liang X., Li P., Deng Y., Pei X., Tan Y., Zhai K., Wang P. (2017). Tough, stretchable chemically cross-linked hydrogel using core–shell polymer microspheres as cross-linking junctions. Polymer.

