# Supplementary material for: Polymerizable Microsphere-Induced High Mechanical Strength of Hydrogel Composed of Acrylamide
Source: Materials (Basel). 2018 May 24;11(6):880. doi: 10.3390/ma11060880 (PMC6025025; doi:10.3390/ma11060880)
Supplement: Supplementary file 1 [file materials-11-00880-s001.pdf]

Supporting Information

Characterization of samples by IR, NMR and SEM

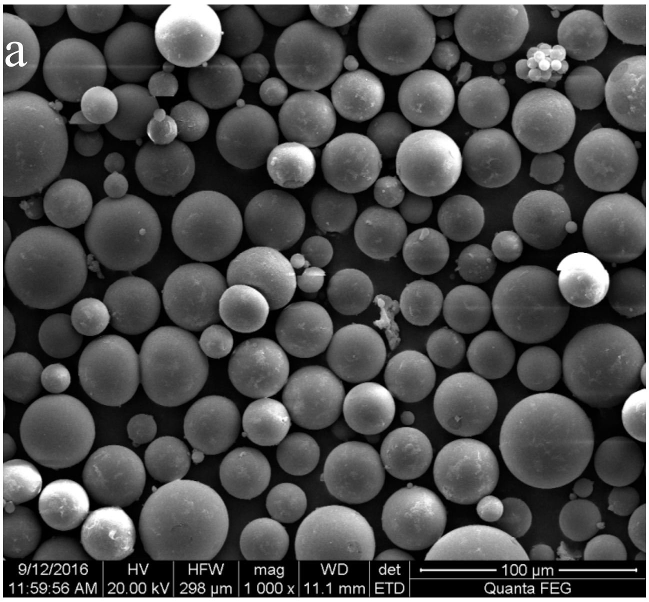

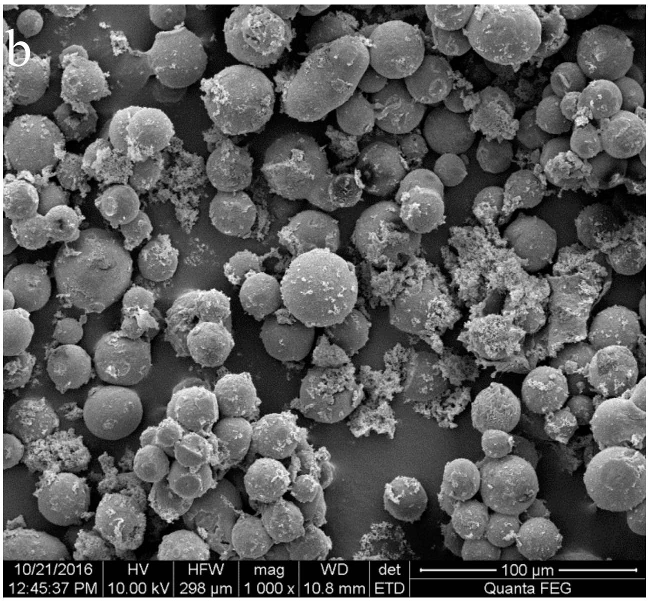

Fig.S1 SEM images of AM/HEMA microspheres (a, ordinary polymeric microspheres; b, polymerizable microspheres)

The microspheres were prepared by KBr tabletting method, and the structure of the microspheres was characterized by 80 vacuum Fu Li ye spectrometer (German Bruker Company). After loading the sample into the nuclear tube, the parameters of the Avance III 500 Nuclear Magnetic Resonance Spectromete (German Bruker Company) were adjusted, and the copolymer microspheres were measured by NMR.

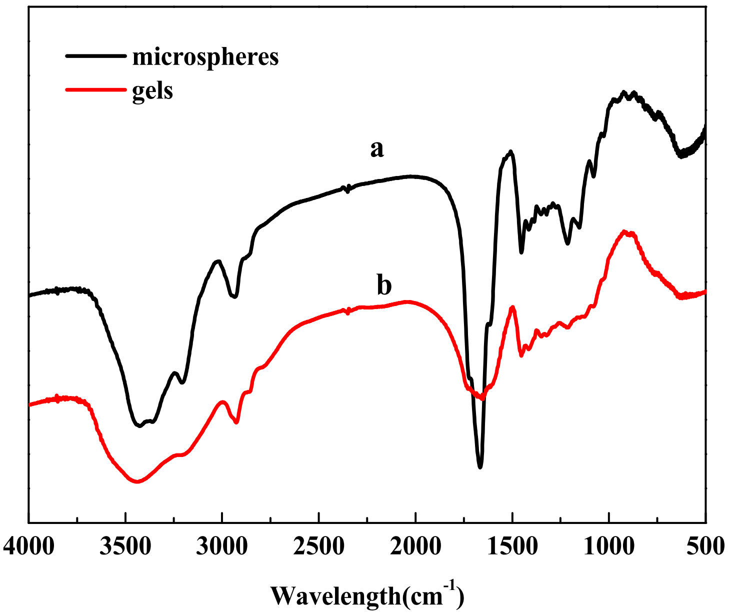

Fig.S2 FT-IR spectra of polymer a) polymerizable AM/HEMA microspheres; b) hydrogel containing polymerizable microspheres

As can be seen from Fig.S2, the bending vibration at the wavelength of 1600cm^{-1} to 1750cm^{-1}absorption peaks attribute to the double bond(C=C) and double bond(C=O), but two peaks overlap. Therefore, it is impossible to determine whether the double bond(C=C) on the surface of the polymerizable microspheres has reacted with acrylamide by infrared spectroscopy. However, the difference was found from the ^{13}C NMR experiments. In contrast to Fig. S3a and Fig. S3b, it is found that in Fig. S3a, characteristic peaks appear at the chemical shift 100ppm, belonging to the double bond(C=C). The characteristic peaks of this functional group disappeared in Fig. S3b Thus it can be seen that the double bond(C=C) originally existed on the surface of the polymerizable microspheres, which have the free radical polymerization with acrylamide under the action of the initiator, thus forming the hydrogels containing polymerizable microspheres.

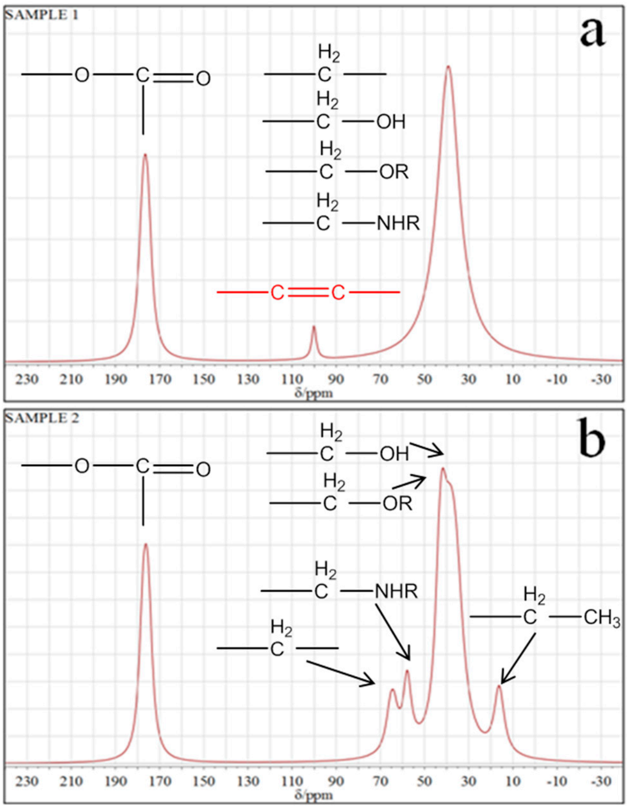

Fig.S3 ^{13}C NMR spectrum of polymer a) polymerizable AM/HEMA microspheres; b) hydrogel containing polymerizable microspheres
